# Clinical outcomes in ER+ HER2 -node-positive breast cancer patients who were treated according to the Recurrence Score results: evidence from a large prospectively designed registry

**DOI:** 10.1038/s41523-017-0033-7

**Published:** 2017-09-08

**Authors:** Salomon M. Stemmer, Mariana Steiner, Shulamith Rizel, David B. Geffen, Bella Nisenbaum, Tamar Peretz, Lior Soussan-Gutman, Avital Bareket-Samish, Kevin Isaacs, Ora Rosengarten, Georgeta Fried, Debbie McCullough, Christer Svedman, Steven Shak, Nicky Liebermann, Noa Ben-Baruch

**Affiliations:** 1 Davidoff Center, Rabin Medical Center, Petah Tikva, Israel; 2 Sackler Faculty of Medicine, Tel Aviv University, Tel Aviv, Israel; 3 Lin Medical Center, Haifa, Israel; 4 Department of Oncology, Soroka University Medical Center and the Faculty of Health Sciences, Ben-Gurion University of the Negev, Beer Sheva, Israel; 5 Oncology Department, Meir Medical Center, Kfar Saba, Israel; 6 Sharett Institute of Oncology, Hadassah-Hebrew University Medical Center, Jerusalem, Israel; 7 Oncotest Division, Teva Pharmaceutical Industries, Ltd., Shoham, Israel; 8 BioInsight Ltd., Zichron Yaakov, Israel; 9 Oncology Department, Ha’emek Medical Center, Afula, Israel; 10 Oncology Institute, Shaare Zedek Medical Center, Jerusalem, Israel; 11 Oncology Department, Rambam Health Care Campus, Haifa, Israel; 12 Genomic Health Inc., Redwood City, CA USA; 13 Community Division, Clalit Health Services, Tel Aviv, Israel; 14 Oncology Department, Kaplan Medical Center, Rehovot, Israel

## Abstract

The Recurrence Score® is increasingly used in node-positive ER+ HER2-negative breast cancer. This retrospective analysis of a prospectively designed registry evaluated treatments/outcomes in node-positive breast cancer patients who were Recurrence Score-tested through Clalit Health Services from 1/2006 through 12/2011 (*N* = 709). Medical records were reviewed to verify treatments/recurrences/survival. Median follow-up, 5.9 years; median age, 62 years; 53.9% grade 2; 69.8% tumors ≤ 2 cm; 84.5% invasive ductal carcinoma; 42.0% N1mi, and 37.2%/15.5%/5.2% with 1/2/3 positive nodes; 53.4% Recurrence Score < 18, 36.4% Recurrence Score 18–30, and 10.2% Recurrence Score ≥ 31. Overall, 26.9% received adjuvant chemotherapy: 7.1%, 39.5%, and 86.1% in the Recurrence Score < 18, 18–30, and ≥ 31 group, respectively. The 5-year Kaplan–Meier estimates for distant recurrence were 3.2%, 6.3%, and 16.9% for these respective groups and the corresponding 5-year breast cancer death estimates were 0.5%, 3.4%, and 5.7%. In Recurrence Score < 18 patients, 5-year distant-recurrence rates for N1mi/1 positive node/2–3 positive nodes were 1.2%/4.4%/5.4%. As patients were not randomized to treatment and treatment decision is heavily influenced by Recurrence Score, analysis of 5-year distant recurrence by chemotherapy use was exploratory and should be interpreted cautiously: In Recurrence Score < 18, recurrence rate was 7.7% in chemotherapy-treated (*n* = 27) and 2.9% in chemotherapy-untreated patients (*n* = 352); *P* = 0.245. In Recurrence Score 18–30, recurrence rate in chemotherapy-treated patients (*n* = 102) was significantly lower than in untreated patients (*n* = 156) (1.0% vs. 9.7% *P* = 0.019); in Recurrence Score ≤ 25 (the RxPONDER study cutoff), recurrence rate was 2.3% in chemotherapy-treated (*n* = 89) and 4.4% in chemotherapy-untreated patients (*n* = 488); *P* = 0.521. In conclusion, our findings support using endocrine therapy alone in ER+ HER2-negative breast cancer patients with micrometastases/1–3 positive nodes and Recurrence Score < 18.

## Genetic testing: Gene panel guides treatment for node-positive patients

Women with breast cancer that has spread to the lymph nodes do well on anti-hormone treatment alone if they score under 18 on OncotypeDX. Salomon Stemmer from Rabin Medical Center in Petah Tikvah, Israel, and colleagues conducted the first analysis of a large prospectively designed registry in which patients with breast cancer cells in the underarm lymph nodes have taken the 21-gene expression analysis known as OncotypeDX to guide their treatment. Among the 709 women with node-positive, ER-positive, HER-negative disease, patients with test scores under 18 did just as well if they received chemotherapy or not in addition to anti-hormone treatment, whereas those with scores of 18 to 30 had significantly lower recurrence rates if they received both therapies. The findings suggest that only women with OncotypeDX scores under 18 can safely forgo chemotherapy.

## Introduction

The 21-gene Recurrence Score®(RS) assay (Oncotype DX® Breast Recurrence Score assay, Genomic Health Inc), is incorporated in major international guidelines,^1–4^ and is widely used to guide treatment decisions in estrogen receptor (ER)+ human epidermal growth factor receptor 2 (HER2)-negative node-negative breast cancer. The prognostic/predictive abilities of the RS assay in this population were first validated using a prospective–retrospective study design.^5–7^ More recently, the RS was further validated as a prognostic indicator in the TAILORx and West German Study Group (WSG) PlanB phase 3 trials in which patients were treated based on their RS results, and in the prospectively designed analysis of the Surveillance, Epidemiology, and End Results (SEER) registry, which was complemented with the RS results.^8–10^ Furthermore, we previously reported findings from an analysis of a prospectively designed registry including 2028 N0/N1mi ER+ breast cancer patients who were tested through Clalit Health Services (CHS, the largest health maintenance organization in Israel),^11^ which complemented the TAILORx, WSG PlanB, and SEER studies by providing both rate of recurrence and breast cancer-specific mortality in a cohort of patients where decision making incorporated the RS in real-life clinical practice.

Withholding chemotherapy in ER+ HER2-negative node-positive breast cancer patients remains controversial even in the presence of data from such studies as the ATAC, ABCSG 6, and ABCSG 8 trials, which have demonstrated that many patients with 1–3 positive nodes do well on endocrine therapy alone.^12–14^ To further help identify patients who can safely forgo chemotherapy, RS testing is increasingly used in patients with up to three positive axillary lymph nodes. Similar to the approach used for the node-negative population, the initial validation of the prognostic/predictive abilities of the RS assay in the node-positive population employed a prospective–retrospective study design.^7, 15^ However, unlike the node-negative population, at present, the additional evidence supporting RS testing in the node-positive population is limited to the aforementioned WSG PlanB study in which all patients with RS > 11 received chemotherapy, and to a secondary analysis of the SEER registry in which only breast cancer-specific mortality has been reported.^9, 10^ Prospective evidence from the phase 3 RxPONDER (SWOG S1007) trial evaluating endocrine therapy alone vs. chemoendocrine therapy in patients with 1–3 positive nodes and RS ≤ 25 is anticipated in the future.^16^

In Israel, CHS approved reimbursement of the RS assay for node-negative patients in 1/2006 and extended its coverage to include node-positive patients in 1/2008. Since its initial reimbursement approval by CHS and through November 2016, 5483 node-negative, and 1850 patients with N1mi/1–3 positive nodes have been tested through CHS. The focus of the current CHS registry analysis is on the latter population. This analysis was designed to evaluate, in real-life clinical practice, the relationship between the RS results, adjuvant treatments received, and clinical outcomes in patients with N1mi or 1–3 positive nodes who were RS-tested more than 5 years ago and had their RS results incorporated into clinical decision making. Notably, this study complements another study focusing on node-negative patients who were RS-tested through CHS.^17^

## Results

### Patient characteristics

Between 1/2006 and 12/2011, 755 CHS members with breast cancer and N1mi or 1–3 positive lymph nodes were RS-tested through CHS. Forty patients were excluded for various reasons (received neoadjuvant treatment, were metastatic at diagnosis, etc). For four additional patients (0.5%) recurrence data were not available or they were lost to follow-up, and two more patients were excluded because they recurred within 6 months of testing (Supplementary Fig. S1). The final cohort included 709 patients and the median follow-up was 5.9 (range, 0.3–10.3) years.

Most patients were female (98%). Median age was 62 (interquartile-range, 53–67) years, 84.6% were ≥50 years; 53.9% had grade 2 tumors; 69.8% had tumors ≤ 2 cm in size, and 84.5% had invasive ductal carcinoma. Less than half of all patients (42.0%) were N1mi, whereas 37.2%, 15.5%, and 5.2% of patients were diagnosed with 1, 2, and 3 positive axillary lymph nodes, respectively (Table 1). Overall, patient characteristics seemed similar in patients across the nodal status groups, and each group had median follow-up >5 years (Supplementary Table S1).

**Table 1:** Baseline patient and tumor characteristics

	All patients
	*N* = 709
Gender, *n* (%)	
Female	695 (98.0)
Male	14 (2.0)
Age	
Median (interquartile range), years	62 (53–67)
Age category, *n* (%)	
<40 years	17 (2.4)
40–49 years	92 (13.0)
50–59 years	195 (27.5)
60–69 years	269 (37.9)
70–79 years	123 (17.3)
≥80 years	13 (1.8)
Tumor size in the greatest dimension	
Median (interquartile range), cm	1.7 (1.3–2.3)
Mean (SD), cm	1.8 (0.92)
Tumor size category, *n* (%)	
≤1 cm	115 (16.2)
>1–2 cm	380 (53.6)
>2	205 (28.9)
Unknown	9 (1.3)
Tumor grade category, *n* (%)	
Grade 1	102 (14.4)
Grade 2	382 (53.9)
Grade 3	113 (15.9)
Not applicable/unknown^a^	112 (15.8)
Histology, *n* (%)	
IDC	599 (84.5)
ILC	85 (12.0)
Papillary	9 (1.3)
Mucinous/colloid	2 (0.3)
Other/unknown	14 (2.0)
Nodal involvement, *n* (%)	
N1mi	298 (42.0)
1 Positive lymph node	264 (37.2)
2 Positive lymph node	110 (15.5)
3 Positive lymph node	37 (5.2)

*IDC* invasive ductal carcinoma, *ILC* invasive lobular carcinoma

^a^ 71% of unknown tumor grade are ILC

### RS distribution and adjuvant chemotherapy use

Overall, 379 patients (53.4%) had RS < 18, 258 (36.4%) had RS 18–30, and 72 (10.2%) had RS ≥ 31. A wide RS distribution was observed within each level of clinicopathological characteristic such as nodal status (N1mi, 1, 2, and 3 positive nodes), age (<40, 40-<50, 50-<60, 60-<70, 70-<80, and ≥80 years), tumor size (≤1,<1–2, and≥ 2 cm), and tumor grade (1–3) (Supplementary Fig. S2).

The use of adjuvant chemotherapy was consistent with the RS result. Overall,chemotherapy use was 26.9%, with 7.1% (27/379), 39.5% (102/258), and 86.1% (62/72) receiving adjuvant chemotherapy in patients with RS < 18, 18–30, and≥31, respectively. Within the group of patients with RS 18–30, adjuvant chemotherapy use tended to increase with increasing RS results (Supplementary Fig. S3).

### Distant recurrence rates

**Fig. 1:**
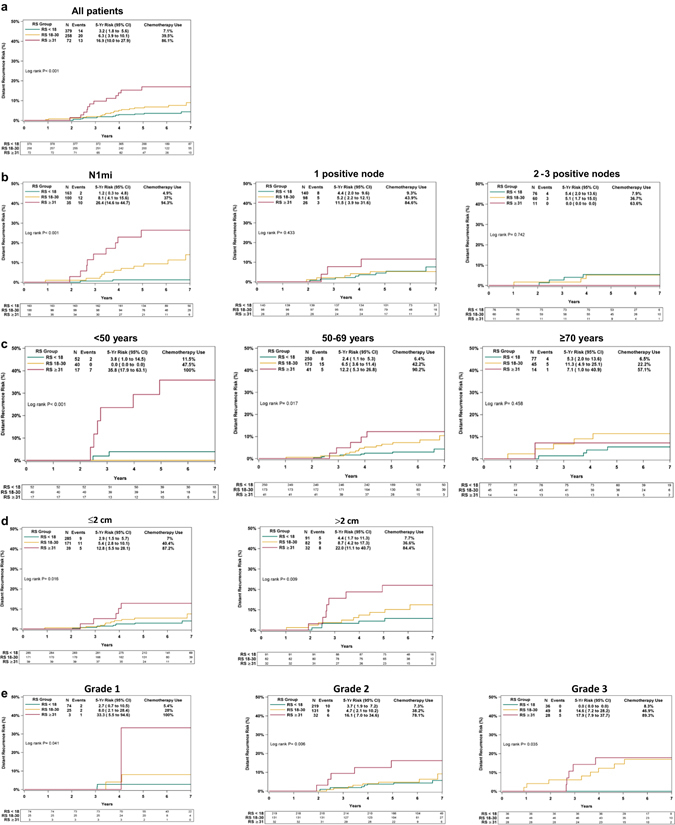
Kaplan–Meier distant recurrence curves by Recurrence Score (*RS*) groups and clinicopathological characteristics. Rates of distant recurrence for the entire cohort (**a**) and by nodal status (**b**), age (**c**) tumor size (**d**), and tumor grade (**e**). For each RS category, the percentage of patients receiving chemotherapy is indicated. The *box* under each graph presents the number of patients at risk at each time point. Results for the subgroup analyses (**b–e**) should be interpreted cautiously due to small number of patients in some of the subgroups, low event rates, and the potential for selection bias with respect to the patients being RS tested

Overall, with a median follow-up of 5.9 years, 47 patients had distant recurrences; 14/379, 20/258, and 13/72 in patients with RS results <18, 18–30, and ≥31, respectively. Kaplan–Meier (KM) estimates for 5-year distant recurrence rates differed significantly between the RS groups (*P* < 0.001; log-rank test). Rates in the RS < 18, 18–30, and ≥31 groups were 3.2% (95% confidence intervals [CI], 1.8–5.6%),6.3% (95% CI, 3.9–10.1%), and 16.9% (95% CI, 10.0–27.9%), respectively (Fig. 1a). Rates in the RS ≤ 25 and RS > 25 groups (RxPONDER categorization) also differed significantly (RS ≤ 25, 4.0% [95% CI, 2.7–6.0%]; RS > 25, 13.1% [95% CI, 8.4–20.3%]; *P* < 0.001; log-rank test) (Supplementary Fig. S4). Figure 1b–e presents subgroup analyses evaluating rates of distant recurrence by nodal status, age, tumor size, and tumor grade. Examining patients with RS < 18 by nodal status demonstrated a 5-year distant recurrence rate of 1.2% (95% CI, 0.3–4.8%), 4.4% (95% CI, 2.0–9.6%), and 5.4% (95% CI, 2.0–13.6%) for N1mi, patients with 1 positive node, and 2–3 positive nodes, respectively (Fig. 1b).

### Breast cancer death rates

**Fig. 2:**
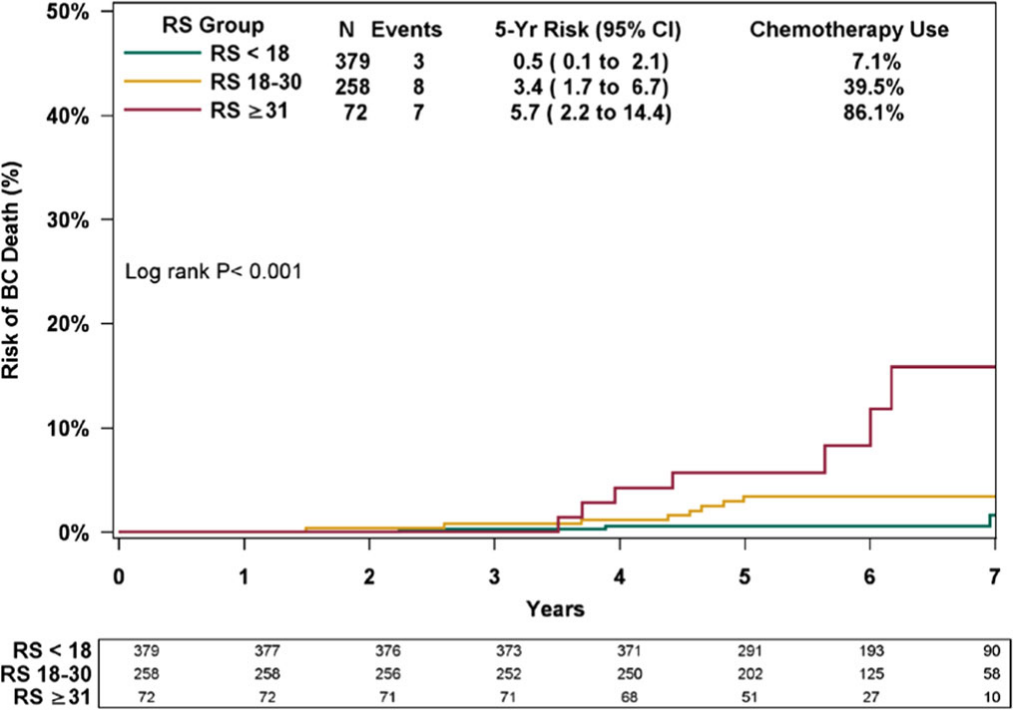
Kaplan–Meier breast cancer death curves by Recurrence Score (*RS*) groups in all patients. The *box* under the curve presents the number of patients at risk at each time point

Overall, 18 breast cancer deaths were documented including 3/379, 8/258, and 7/72 in patients with RS < 18, RS 18–30, and RS ≥ 31, respectively. KM estimates for 5-year breast cancer death rates varied significantly between the RS groups (*P < *0.001; log-rank test; Fig. 2), ranging from 0.5% (95% CI, 0.1–2.1%) in RS < 18 patients, 3.4% (95% CI, 1.7–6.7%) in RS 18–30 patients, and 5.7% (95% CI, 2.2–14.4%) in RS ≥ 31 patients. A significant difference in breast cancer death rates was also observed between RS ≤ 25 and RS > 25 patients (RxPONDER categorization): RS ≤ 25, 1.1% (95% CI, 0.5–2.5%); RS > 25, 6.5% (95% CI, 3.3–12.6%); *P* < 0.001; log-rank test (Supplementary Fig. S4).

### Risk of distant recurrence/breast cancer death in chemotherapy-untreated patients

**Fig. 3:**
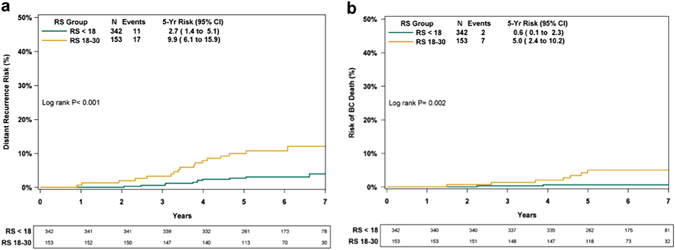
Kaplan–Meier distant recurrence (**a**) and breast cancer death (**b**) curves in chemotherapy-untreated patients with Recurrence Score (*RS*) < 18 and RS 18–30 who received hormone therapy. The *box* under each graph presents the number of patients at risk at each time point

RS testing was developed to predict distant recurrence risk in patients receiving endocrine therapy alone. In addition to not receiving chemotherapy, a small subset of patients (2.5%) did not receive endocrine therapy for various reasons. Therefore, we performed an analysis in the 90.2% of patients in the RS < 18 group and the 59.3% of patients in the RS 18–30 group that received endocrine therapy alone. The KM 5-year distant recurrence rates in these patients were 2.7% (95% CI, 1.4–5.1%) and 9.9% (95% CI, 6.1–15.9%) in the RS < 18 and RS 18–30 groups, respectively (*P* < 0.001; log-rank test; Fig. 3a). The corresponding breast cancer death rates were 0.6% (95% CI, 0.1–2.3%) and 5.0% (95% CI, 2.4–10.2%) (*P* = 0.002; log-rank test; Fig. 3b).

### Risk of distant recurrence by chemotherapy treatment

**Fig. 4:**
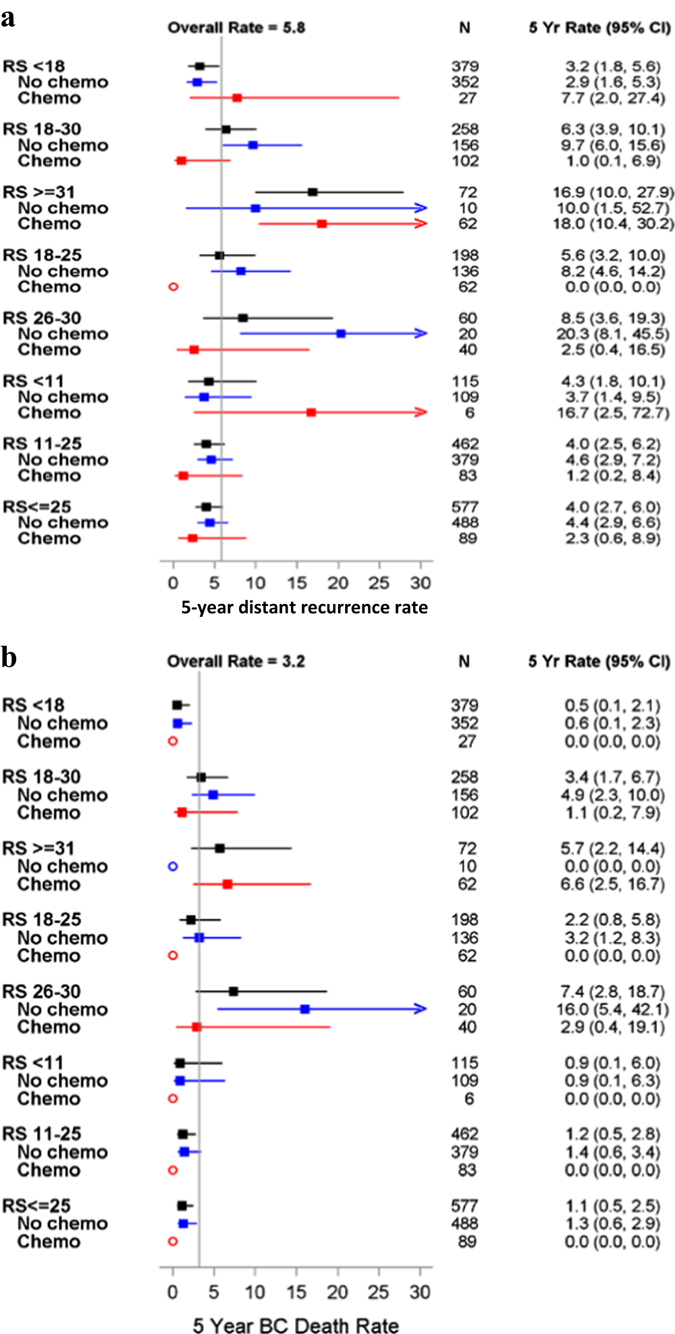
Forest plots of 5-year distant recurrence risk (**a**) and 5-year breast cancer death (**b**) by adjuvant chemotherapy treatment

We assessed the KM estimates for 5-year distant recurrence in patients with RS ≤ 30 by adjuvant chemotherapy use (Fig. 4a). For this analysis, we used the Paik et al.^5^ categorization (<18, 18–30,≥31), as well as RS value of 25 as a cutoff (in alignment with RxPONDER trial^16^). In RS < 18 patients, recurrence rate was 7.7% in chemotherapy-treated (*n* = 27) compared to 2.9% in chemotherapy-untreated patients (*n* = 352); *P = *0.245. In RS 18–30 patients, however, recurrence rate in chemotherapy-treated patients (*n* = 102) was significantly lower than in untreated patients (*n* = 156) (1.0% vs. 9.7%, *P* = 0.019). It appears that this difference stems from the subgroup of RS 26–30 and not from the 18–25 subgroup (*P*-values of 0.017 and 0.058, respectively) (Fig. 4a). In RS ≤ 25 patients (the RS range where RxPONDER patients were randomized), recurrence rate was 2.3% in chemotherapy-treated (*n* = 89) compared to 4.4% in chemotherapy-untreated patients (*n* = 488); *P = *0.521. Analysis of KM estimates for 5-year breast cancer death revealed consistent findings (Fig. 4b). Importantly, as patients were not randomized to treatment, and the number of recurrences/deaths was small, these results should be interpreted very cautiously.

### Univariate and multivariable analyses

In a univariate analysis performed on the entire cohort, tumor size (≥2 vs.<2 cm) and the RS group (<18 vs. ≥31; 18–30 vs. ≥31) were found to have a significant association with distant recurrence risk, whereas age, grade, and nodal status were nonsignificant parameters (Supplementary Table S2). Although nodal status was found to be nonsignificant, we decided to include it in a multivariable regression analysis (on the same cohort) for clinical relevance. Thus, the multivariable analysis included tumor size (≥2 vs.<2 cm), nodal status (N1mi vs.1–3 positive nodes), and the RS group (18–30 vs.<18 and ≥31 vs.<18). Only tumor size and RS group, but not nodal status were found to be significantly associated with distant recurrence risk (Table 2). When age and tumor grade were included in the model, only the RS group and tumor size remained statistically significant (*P = *0.003 and 0.02, respectively). When tumor size and RS were included in the model as continuous parameters, they remained statistically significant (*P < *0.0001 and *P* = 0.006, respectively).

**Table 2:** Multivariable analysis on the entire cohort (chemotherapy-treated and untreated). The analysis evaluated the association between the variables and distant recurrence

Variable^a^	Comparison	Hazard ratio	*P*-value
		(95% confidence intervals)	
Size	≥2 vs. <2 cm	1.8 (1.0–3.3)	0.04
Nodal status	1–3 positive nodes vs. N1mi	0.86 (0.48–1.6)	0.63
RS Group	<18 vs. ≥31	0.23 (0.11–0.50)	0.001
	18–30 vs. ≥31	0.42 (0.20–0.86)	

*RS* Recurrence Score

^a^ The RS group remained a significant variable (*P* < 0.001) when age and tumor grade were also included in the multivariable model

## Discussion

This is the first analysis of a large prospectively designed registry investigating both distant recurrence and breast cancer death rates in patients with N1mi (42% in our cohort) or 1–3 positive nodes (58% in our cohort) where the RS has been incorporated into real-life clinical decision making. We found that chemotherapy use was consistent with the RS, and that clinical outcomes were very good in patients with RS < 18 (5-year distant recurrence rate, 3.2%; 5-year breast cancer death rate, 0.5%) and also excellent in the 90.2% of patients with RS < 18 selected to receive endocrine therapy alone (5-year distant recurrence rate, 2.7%; 5-year breast cancer death rate, 0.6%).

Chemotherapy use was aligned with the RS results. This finding is consistent with the CHS N0/N1mi analysis and with decision impact studies conducted in the N1mi/N1 population worldwide.^18–27^ Within the intermediate RS category (RS 18–30), chemotherapy use increased with higher RS results, consistent with prior Israeli studies including the analysis of the CHS N0/N1mi cohort and with the SEER registry analysis for node-negative and node-positive patients.^11, 28, 29^ This observation suggests that clinicians assess the RS result as a continuous parameter.

Our findings are consistent with those found in the original prospective–retrospective validation studies in node-positive patients, with the analysis of node-positive patients in the SEER registry, and with the results of the prospective WSG PlanB study, which included node positive and high-risk node-negative patients, and as such constitutes the only prospective evidence for the clinical outcomes following RS-based treatment in node-positive patients (41.2% of the study patients with available tissue within the tumor bank).^7, 9, 10, 15^ In WSG PlanB, the RS risk groups were RS ≤ 11, 12–25, and>25 (similar to those in TAILORx in which the lower range was RS ≤ 10).^8, 9^ Patients in the lower RS range were recommended to omit chemotherapy, whereas all patients with RS ≥ 12 received chemotherapy. After median follow-up of 35 months, 3-year disease-free survival in patients with RS ≤ 11 (chemotherapy untreated, *n* = 348) was 98% vs. 98% and 92% in those with RS 12–25 and RS > 25, respectively.^9^ Distant recurrence rates in WSG PlanB patients have not been reported yet.^9^ Our findings are also consistent with the previously reported analysis of the CHS N0/N1mi cohort,^11^ as in both cases, chemotherapy use was aligned with the RS results and the risk of recurrence was significantly higher in patients with higher RS results. Not surprisingly, in the RS < 18 and RS 18–30 groups, chemotherapy use was higher in the current cohort than in the corresponding RS groups in the CHS N0/N1mi cohort, and the 5-year risk of distant recurrence was also higher in each of the RS groups (RS < 18, 0.8% vs. 3.2%; RS 18–30, 3.2% vs. 6.3%; RS ≥ 31, 10.3% vs. 16.9%). Notably, in the original validation study in node-positive patients (SWOG 8814), all patients received anthracycline-based chemotherapy; whereas, in WSG Plan B, patients received anthracycline-based or taxane-based chemotherapy.^9, 15^ In our analysis, as in the previously reported analysis of the N0/N1mi CHS cohort,^11^ the chemotherapy used included predominantly anthracycline-based or taxane-based regimens.

Thus far, MINDACT has been the only prospective phase 3 trial reporting findings from randomized arms in which a genomic assay (the 70-gene signature, MammaPrint® by Agendia, Inc) was investigated as an aid to treatment decisions in early-stage breast cancer.^30^ This trial enrolled 6693 patients with early-stage breast cancer (79% node-negative and 21% node-positive), of which 644 qualified for the primary analysis. The trial met its primary endpoint as patients who had high risk by clinical characteristics (using Adjuvant!Online) and low risk by the 70-gene signature and were randomized to the non-chemotherapy arm had a 5-year rate of distant recurrence-free survival of 94.7% (the lower boundary of the 95% CI was 92.5%, which is above the predefined boundary of 92%).^30^ Notably, in patients (per-protocol population) with high clinical risk and low risk by the 70-gene signature, disease-free survival was significantly better with chemotherapy (93.3% vs. 90.3%, *P* = 0.03). In patients (per-protocol population) with low clinical risk and high risk by the 70-gene signature, no significant chemotherapy benefit was observed.^30^ In contrast to the MINDACT findings, our results (consistent with the prospective-retrospective RS validation studies showing minimal chemotherapy benefit in low RS patients^15^) strongly support forgoing chemotherapy in patients with ER+ HER2-negative breast cancer with micrometastases/1–3 positive nodes and RS < 18 and treating them with endocrine therapy alone. Unlike our previously reported findings for N0 patients,^11^ the current analysis of node-positive patients does not support sparing adjuvant chemotherapy in patients with RS 18–25 and suggests that chemotherapy treatment should be recommendedto all patients with RS > 18.

Notably, in this analysis of a real-life, national level large registry, no exclusion criteria were applied with respect to age, gender, location, socioeconomic status or comorbidities. Nevertheless, the registry analysis is limited by its nonrandomized design and the potential for selection bias with respect to patients being selected for RS testing; in Israel the test is less frequently used in the node-positive population relative to the node-negative population and we estimate that 40% of eligible N1mi/N1 patients are being tested. Another limitation is the generalizability of our findings, as the population in our analysis was mostly Caucasian. As chemotherapy treatment decisions at CHS rely heavily on the RS result, particularly within the RS < 18 and RS ≥ 31 groups, analyses of outcomes by chemotherapy use should be interpreted cautiously. The subgroup analyses should also be interpreted cautiously as some of the groups (e.g., patients with 2 or 3 positive nodes) are small and the event rate is very low. Notably, if the event rate is this low in the RxPONDER trial, where the analysis is event-driven, it may take longer than expected until the data become available. Chemotherapy benefit is expected to be seen in the first years after treatment and our cohort has a median follow-up of 5.9 years. Further analysis is planned when the cohort has longer median follow-up to assess risk of late recurrences.

In conclusion, in this analysis of prospectively-designed registry assessing distant recurrence and breast cancer death in ER+ HER2-negative breast cancer patients with N1mi or 1–3 positive nodes where the RS result has been incorporated in treatment decision making, chemotherapy-untreated patients with RS < 18 had very good clinical outcomes supporting the use of endocrine therapy alone in this population.

## Methods

### Patient population

This retrospective analysis of a prospectively designed registry investigated the relationship between the RS, adjuvant treatments received, and clinical outcomes in all CHS patients with ER+ HER2-negative breast cancer and N1mi or 1–3 positive nodes who were RS-tested between 1/2006 (CHS approval of the assay) and 12/2011. The primary endpoint was distant recurrence and the secondary endpoint was breast cancer death. Exclusion criteria included: ER-negativity by immunohistochemistry (IHC; cutoff: 0.5) and reverse transcription polymerase chain reaction (RT-PCR) performed as part of the 21-gene assay (cutoff: 6.5 units); HER2 positivity by IHC (cutoff: 3), fluorescence in situ hybridization (ratio cutoff: 2.2), or RT-PCR performed as part of the 21-gene assay (cutoff: 10.7 units); receiving trastuzumab in the adjuvant setting; receiving neoadjuvant treatment; having metastatic disease at the time of testing or within 6 months of testing (as the latter were assumed to be metastatic at diagnosis); and receiving adjuvant therapy for another malignancy within 6 months of testing.

This registry analysis was approved by the institutional review boards of the CHS Community Division and participating medical centers, and was granted a waiver for obtaining patient consent. It was conducted in accordance with the Declaration of Helsinki.

### Data source

The following data sources were used: The Teva Pharmaceutical Industries Oncotest database (for RS results and patient/tumor characteristics); medical records (for treatments received and recurrence/death status); and the CHS claims arm (for treatments received and death status). Those extracting the clinical information were unaware of the RS status.

### Statistical analysis

The statistical analysis plan was pre-specified. Descriptive statistics were used to summarize clinicopathological characteristics and adjuvant treatment decision. The primary endpoint was 5-year KM distant recurrence estimates and 95% CI by RS risk categories as defined by Paik et al.^5^ (<18, 18–30,≥31). Patients without recurrence were censored at the time of last follow-up, date of medical records review, or at time of death (due to any cause). A secondary endpoint was 5-year KM breast cancer death estimates and 95% CI. Patients with metastatic disease at the time of death were considered events, patients were censored at the time of last follow-up or death from other causes, and recurrences were ignored for purposes of this endpoint. Analysis was also performed on clinicopathological subgroups, as well as within the intermediate RS group (18–25, 26–30), using a cutoff of 25 as in the RxPONDER trial.^16^ As exploratory analysis, 5-year KM distant recurrence estimates and 95% CI by chemotherapy use within RS risk categories as defined by Paik et al.^5^ and within the intermediate group (18–25, 26–30) were examined. A 2-degrees of freedom log-rank test was used to compare distant recurrence rates across RS groups. Survival curves are presented through 7-years, though all observed events and follow-up are utilized for statistical tests. Cox proportional hazards regression models were used to evaluate the association of RS group (<18, 18–30, ≥31), age (50–69 vs. <50; ≥70 vs. <50 years), size (<2, ≥2 cm), grade (1–3), and nodal status (N1mi vs. 1–3 positive nodes) with distant recurrence. RS and tumor size were also included as continuous variables. Univariate analysis identified a set of prognostic baseline factors, and a full multivariable model was fit. Non-significant covariates were removed from the multivariate analysis. Proportional hazards assumptions were assessed and met in the final model. SAS 9.4 (SAS Institute Inc., Cary, NC) was used for the analysis. *P* < 0.05 was considered statistically significant.

### Data availability

All relevant data are available upon request from the corresponding author.

## Electronic supplementary material

Supplemental materials
