# Supplementary material for: Clinical outcomes in ER+ HER2 -node-positive breast cancer patients who were treated according to the Recurrence Score results: evidence from a large prospectively designed registry
Source: NPJ Breast Cancer. 2017 Sep 8;3:32. doi: 10.1038/s41523-017-0033-7 (PMC5591314; doi:10.1038/s41523-017-0033-7)
Supplement: Supplementary file 1 — Supplemental materials [file 41523_2017_33_MOESM1_ESM.docx]

**SUPPLEMENTARY INFORMATION**

**Table S1**. Baseline Patient and Tumor Characteristics by Nodal Status

|  | All patients  *N* = 709 | N1mi  *N* = 298 | 1 positive lymph node  *N* = 264 | 2 positive lymph node  *N* = 110 | 3 positive lymph node  *N* = 37 |
| --- | --- | --- | --- | --- | --- |
| Median follow-up, years | 5.9 | 6.0 | 6.0 | 5.7 | 5.4 |
| Gender, *n* (%) |  |  |  |  |  |
| Female | 695 (98.0) | 294 (98.7) | 257 (97.4) | 107 (97.3) | 37 (100.0) |
| Male | 14 (2.0) | 4 (1.3) | 7 (2.6) | 3 (2.7) | 0 (0.0) |
| Age |  |  |  |  |  |
| Median (interquartile range), years | 62 (53-67) | 59 (51-66) | 62 (54-67) | 63 (56-69) | 66 (58-74) |
| Age category, *n* (%) |  |  |  |  |  |
| <40 years | 17 (2.4) | 10 (3.4) | 6 (2.3) | 1 (0.9) | 0 (0.0) |
| 40-49 years | 92 (13.0) | 53 (17.8) | 31 (11.7) | 6 (5.5) | 2 (5.4) |
| 50-59 years | 195 (27.5) | 87 (29.2) | 66 (25.0) | 33 (30.0) | 9 (24.3) |
| 60-69 years | 269 (37.9) | 102 (34.2) | 108 (40.9) | 48 (43.6) | 11 (29.7) |
| 70-79 years | 123 (17.3) | 43 (14.4) | 48 (18.2) | 19 (17.3) | 13 (35.1) |
| ≥80 years | 13 (1.8) | 3 (1.0) | 5 (1.9) | 3 (2.7) | 2 (5.4) |
| Tumor size in the greatest dimension |  |  |  |  |  |
| Median (interquartile range), cm | 1.7 (1.3-2.3) | 1.6 (1.2-2.1) | 1.7 (1.2-2.4) | 1.9 (1.3-2.3) | 2.0 (1.5-2.5) |
| Mean (SD), cm | 1.8 (0.92) | 1.8 (0.86) | 1.9 (1.02) | 2.0 (0.92) | 1.9 (0.70) |
| Tumor size category, *n* (%) |  |  |  |  |  |
| ≤1 cm | 115 (16.2) | 48 (16.1) | 45 (17.0) | 17 (15.5) | 5 (13.5) |
| >1 - 2 cm | 380 (53.6) | 172 (57.7) | 135 (51.1) | 53 (48.2) | 20 (54.1) |
| >2 | 205 (28.9) | 74 (24.8) | 79 (29.9) | 40 (36.4) | 12 (32.4) |
| Unknown | 9 (1.3) | 4 (1.3) | 5 (1.9) | 0 (0.0) | 0 (0.0) |
| Tumor grade category, *n* (%) |  |  |  |  |  |
| Grade 1 | 102 (14.4) | 43 (14.4) | 41 (15.5) | 14 (12.7) | 4 (10.8) |
| Grade 2 | 382 (53.9) | 157 (52.7) | 141 (53.4) | 63 (57.3) | 21 (56.8) |
| Grade 3 | 113 (15.9) | 50 (16.8) | 40 (15.2) | 16 (14.5) | 7 (18.9) |
| Not applicable/Unknown | 112 (15.8) | 48 (16.1) | 42 (15.9) | 17 (15.5) | 5 (13.5) |
| Histology, *n* (%) |  |  |  |  |  |
| IDC | 599 (84.5) | 248 (83.2) | 225 (85.2) | 93 (84.5) | 33 (89.2) |
| ILC | 85 (12.0) | 38 (12.8) | 28 (10.6) | 15 (13.6) | 4 (10.8) |
| Papillary | 9 (1.3) | 4 (1.3) | 5 (1.9) | 0 (0.0) | 0 (0.0) |
| Mucinous/colloid | 2 (0.3) | 2 (0.7) | 0 (0.0) | 0 (0.0) | 0 (0.0) |
| Other/unknown | 14 (2.0) | 6 (2.0) | 6 (2.3) | 2 (1.8) | 0 (0.0) |
| Recurrence Score group, *n* (%) |  |  |  |  |  |
| Low (<18) | 379 (53.4) | 163 (54.7) | 140 (53.0) | 58 (52.7) | 18 (48.6) |
| Intermediate (18-30) | 258 (36.4) | 100 (33.6) | 98 (37.1) | 43 (39.1) | 17 (45.9) |
| High (≥31) | 72 (10.2) | 35 (11.7) | 26 (9.8) | 9 (8.2) | 2 (5.4) |

Abbreviations: IDC, invasive ductal carcinoma; ILC, invasive lobular carcinoma.

**Table S2**. Univariate analysis on the entire cohort (chemotherapy-treated and untreated). The analysis evaluated the association between the variables and distant recurrence.

| **Variable** | **Comparison** | **Hazard Ratio**  **(95% confidence intervals)** | ***P*-value** |
| --- | --- | --- | --- |
| **Age** | 50-69 versus <50 years | 0.76 (0.36-1.6) | 0.70 |
|  | ≥70 versus <50 years | 0.95 (0.38-2.3) |  |
| **Size** | ≥2 versus <2cm | 2.17 (1.2-3.9) | 0.01 |
| **Grade** | 1 versus 3 | 0.40 (0.14-1.1) | 0.13 |
|  | 2 versus 3 | 0.54 (0.28-1.1) |  |
| **Nodes** | 1-3 positive nodes versus N1mi | 0.74 (0.43-1.31) | 0.30 |
| **RS group** | <18 versus ≥31 | 0.19 (0.09-0.40) | <0.001 |
|  | 18-30 versus ≥31 | 0.39 (0.20-0.79) |  |

Abbreviation: RS, recurrence Score.

**Figure S1.**Patient disposition

*N*= 40, excluded not meeting inclusion/exclusion:

*n* = 13, ≥4 positive nodes or unknown number of positive nodes

*n* = 10, received neoadjuvant treatment

*n* = 6, unconfirmed cancer diagnosis

*n* = 5, received trastuzumab in the adjuvant setting

*n* = 3, unknown or positive HER2 status

*n* = 3, metastatic at diagnosis

*N*=2, excluded for recurrence within 6 months of diagnosis (RS of 14, 20)

*N*=755

*N*= 715

*N*=711

*N*=709

*N*=4, excluded for lack of data or were lost to follow up

**Figure S2. Recurrence Score (RS) distribution by clinical and pathological characteristics**. RS distribution by nodal status (**a**), age group (**b**), tumor size (**c**), and tumor grade (**d**). Tumor size information was missing for 9 patients; tumor grade information was not available for 112 patients (71 % of whom had invasive lobular carcinoma).Green, RS<18; yellow, RS: 18-30; red, RS≥31.

| **a**  **** | **b**  **** |
| --- | --- |
| **c**  **** | **d**  **** |

**Figure S3. Adjuvant chemotherapy use in patients with Recurrence Score (RS) 18-30 (*n* = 258).** Number of patients per RS result by chemotherapy use is displayed in the bar chart segments.

**
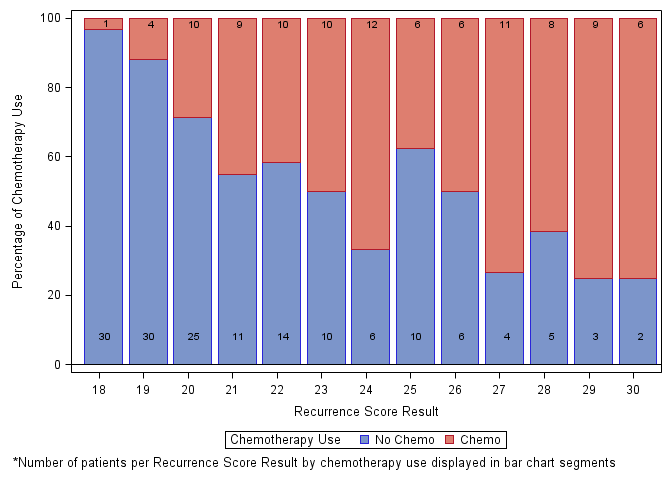
**

**Figure S4.** Kaplan-Meier distant recurrence (**a**) and breast cancer death (**b**) curves by Recurrence Score (RS) groups using the RxPONDER categorization. The box under each graph presents the number of patients at risk at each time point.

| **a**  **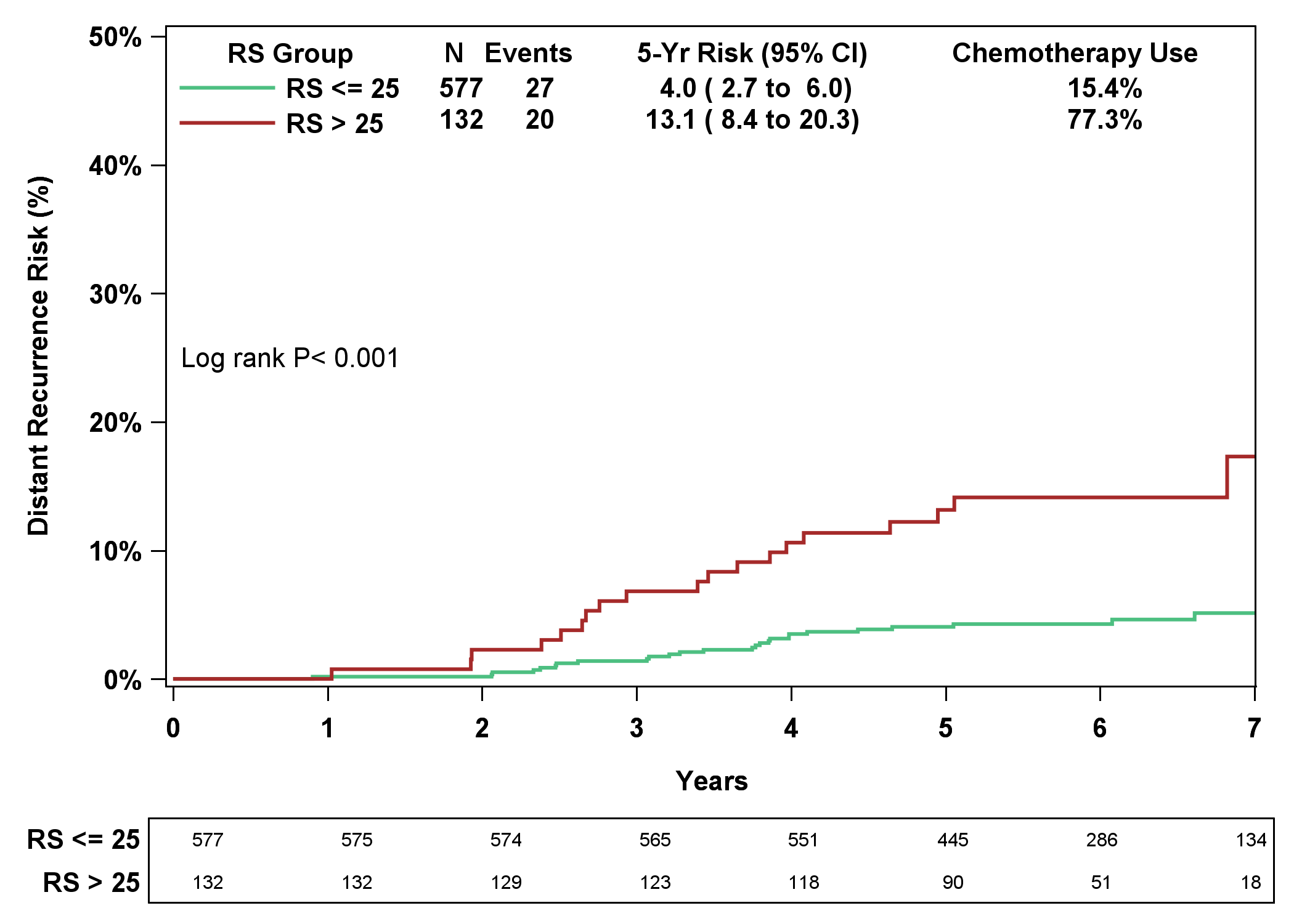** |
| --- |
| **b**  **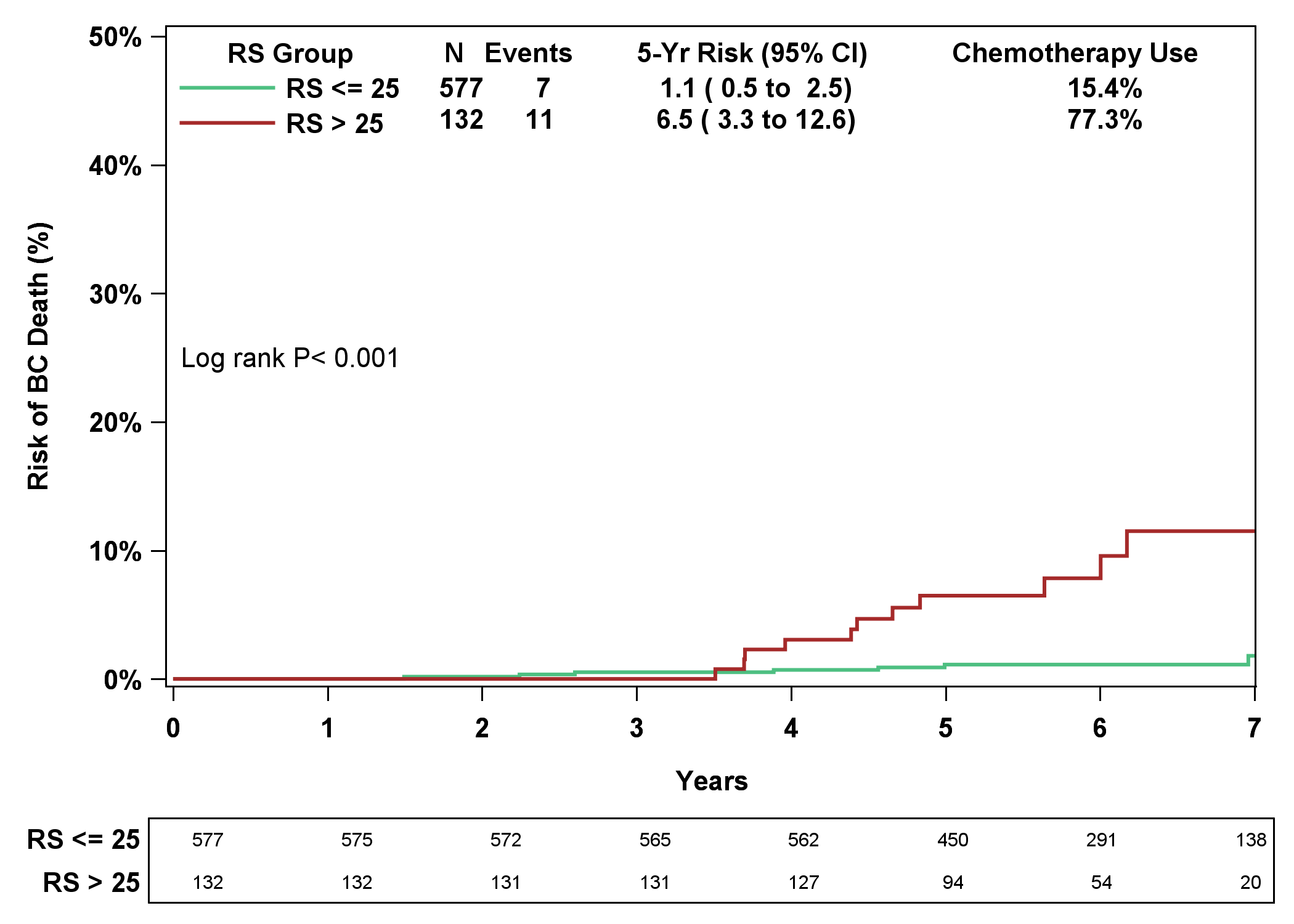** |

Log-rank *P* values were calculated from all of the data.
